# Impact of Driver Mutations on Metastasis-Free Survival in Uveal Melanoma: A Meta-Analysis

**DOI:** 10.3390/cancers16142510

**Published:** 2024-07-10

**Authors:** David Lamas-Francis, Carmen Antía Rodríguez-Fernández, Elia de Esteban-Maciñeira, Paula Silva-Rodríguez, María Pardo, Manuel Bande-Rodríguez, María José Blanco-Teijeiro

**Affiliations:** 1 Department of Ophthalmology, University Hospital of Santiago de Compostela, 15706 Santiago de Compostela, Spain; elia.de.esteban.macineira@sergas.es (E.d.E.-M.); manuel.francisco.bande.rodriguez@sergas.es (M.B.-R.); maria.jose.blanco.teijeiro@sergas.es (M.J.B.-T.); 2 Department of Ophthalmology, Vall d’Hebron University Hospital, 08035 Barcelona, Spain; carmenantia@gmail.com; 3 FarmaChusLab Group, Health Research Institute of Santiago de Compostela (FIDIS), 15706 Santiago de Compostela, Spain; 4 Fundación Pública Galega de Medicina Xenómica, 15706 Santiago de Compostela, Spain; paula.silva.rodriguez@rai.usc.es; 5 Translational Ophthalmology Group, Health Research of Santiago de Compostela (IDIS), 15706 Santiago de Compostela, Spain; 6 Obesidomics Group, Health Research Institute of Santiago de Compostela (IDIS), 15706 Santiago de Compostela, Spain; maruxapardo@hotmail.com

**Keywords:** uveal melanoma, driver mutations, survival, *BAP1*, *GNAQ*, *GNA11*, *SF3B1*

## Abstract

**Simple Summary:**

Certain genetic changes, called driver mutations, can affect how uveal melanoma progresses and spreads. We reviewed and combined data from 13 studies to better understand these effects. A mutation in the *BAP1* gene significantly increases the risk of metastatic disease. Other mutations in *GNAQ*, *GNA11*, or *SF3B1* did not show a similar risk.

**Abstract:**

The prognosis of uveal melanoma is significantly influenced by the risk of metastasis, which varies according to clinical and genetic features. Driver mutations can predict the likelihood of disease progression and survival, although the data in the literature are inconsistent. This meta-analysis aimed to evaluate the prognostic significance of driver mutations, including *GNAQ*, *GNA11*, *BAP1*, and *SF3B1*, in the advancement of uveal melanoma. A comprehensive search of databases yielded relevant studies, and data from 13 studies (848 eyes) were synthesized to assess the impact of these mutations on metastasis-free survival. The *BAP1* mutation and negative immunohistochemistry were associated with a higher risk of metastasis (logHR = 1.44, 95% CI 1.05–1.83). *GNAQ*, *GNA11*, and *SF3B1* mutations did not show a significant increase in risk. In summary, *BAP1* has proven to reliably predict the likelihood of disease progression in uveal melanoma, while further studies are needed to establish the significance of other driver mutations.

## 1. Introduction

Uveal melanoma (UM) is the most prevalent primary malignant intraocular tumor among adults, affecting the choroid in 90% of cases and typically presenting in one eye. The annual incidence in Europe and the United States is approximately 6 cases per million people per year [1]. Although treatment for the primary tumor is generally effective, about half of the patients with UM will eventually develop metastatic disease, usually spreading to the liver in over 90% of cases [2]. Metastasis is the leading cause of death, highlighting the urgent need for therapies to prevent and treat this condition, which has mortality rates exceeding 50% within 5 years [3].

The risk of metastatic disease can be assessed by evaluating various clinical, histopathologic, and genetic features. The TNM staging, the standard prognostic tool for choroidal and ciliary body UM, considers clinical factors such as basal diameter, tumor thickness, ciliary body involvement, and extraocular extension [4]. The larger the primary UM, the greater the number of mutant cells produced. However, histological and genetic factors, which are also crucial, are not included in this staging system. Histopathological indicators of malignancy include epithelioid cell type, a high number of mitoses, increased lymphocyte or macrophage count, and specific extravascular matrix patterns [5,6]. By 1996, a strong association was observed between the loss of one copy of chromosome 3 and the progression to metastatic disease [7]. Subsequent genetic research confirmed that chromosomal abnormalities, such as monosomy 3, 8q gain, and a loss in 1p, are linked to a higher risk of metastasis and a poor prognosis, while a gain in 6p is associated with a favorable prognosis [8].

There has been increasing attention on the impact of activating genetic mutations in *GNAQ* and *GNA11*, loss-of-function mutations in the tumor suppressor gene *BAP1*, and recurrent mutations at codon 625 of *SF3B1* on the advancement of UM towards metastatic disease [8]. *GNAQ* and *GNA11*, essential elements of the G protein-coupled receptor signaling pathway, have received attention for their potential role in the early stages of cancer development and their inconsistent link to prognosis in different studies. Similarly, mutations in *BAP1* and *SF3B1* have exhibited complex relationships, with varying findings regarding their influence on metastasis-free survival.

Given the inconsistencies in the literature, we conducted a meta-analysis to comprehensively evaluate the prognostic significance of these driver mutations in UM progression toward metastatic disease. Our aim is to provide a robust assessment of the impact of *BAP1*, *GNAQ*, *GNA11*, *EIF1AX*, and *SF3B1* mutations on metastasis-free survival in patients with UM, thus informing clinical decision-making and potentially guiding future therapeutic strategies.

## 2. Methods

### 2.1. Data Source and Search Methods

Two authors (DL and ED) searched PubMed for primary research papers published between January 2009 and April 2024 related to driver mutations and UM. The following search strategy was used on 14 April 2024: (“Uveal melanoma” OR “Ocular melanoma” OR “Eye melanoma”) AND (“*GNAQ*” OR “*GNA11*” OR “*PLCB4*” OR “*BAP1*” OR “*SF3B1*” OR “*EIF1AX*”).

### 2.2. Study Selection

Retrospective case series, which included data on metastasis-free survival (MFS) in patients with mutations of *BAP1*, *GNA11*, *GNAQ*, *SF3B1*, and/or *E1FAX*, were included. The risk of metastasis was expressed as Hazard Ratio (HR). Exclusion criteria were as follows: studies providing only other survival measures (such as overall survival or disease-specific death), risk of metastasis expressed as relative risk or odds ratio, studies on animal models, languages different than English or Spanish, literature reviews, and comments on previous studies. This study followed the Preferred Reporting Items for Systematic Reviews and Meta-analysis (PRISMA) guidelines [9] and was in accordance with the Declaration of Helsinki. The study protocol was not registered. No new data were created or analyzed in this study. Data sharing is not applicable to this article.

### 2.3. Data Extraction and Outcomes

The titles and abstracts were screened using the web application Rayyan following the initial search [10]. Two authors, DL and ED, independently reviewed the abstracts to confirm they met the eligibility criteria. Full-text versions of eligible articles were assessed by both authors to select the studies for inclusion in the analysis. Any discrepancies were resolved by discussion between the authors.

The following variables were extracted from the selected studies:

- Study characteristics: year of publication, sample size, mean follow-up, genetic mutations, patients tested for each mutation, the molecular test used, the sample used for analysis (enucleation or biopsy), and survival analysis (overall survival, disease-specific death or metastasis-free survival) including hazard ratio for each variable;
- Primary outcome: metastasis-free survival in patients with UM with *BAP1*, *GNA11*, *GNAQ*, *SF3B1*, or *E1FAX* mutations. Hazard ratios from univariate models and 95% CI values were obtained from the main manuscript or Supplementary Materials when available. When hazard ratios were not presented, but there were sufficient data in Kaplan–Meier diagrams, these were estimated by comparing failure rates between the mutated and wild-type groups at different time intervals, and failure rates were found by dividing the number of events by the number of individuals at risk at the start of each interval [11]. The results from the Silva et al. study were obtained directly from the initial data following a reanalysis.

### 2.4. Assessment of Risk of Bias and Quality of Studies

Two authors (DL and ED) independently evaluated the risk of bias using the Newcastle–Ottawa scale for non-randomized studies, resolving any discrepancies through discussion. The methodological quality of the studies was examined using the Strengthening the Reporting of Observational Studies in Epidemiology (STROBE) Statement. To assess publication bias, we employed funnel and Galbraith plots, along with regression tests for asymmetry in funnel plots (Supplementary Figures S1–S8).

### 2.5. Data Synthesis and Analysis

For the statistical analysis of this study, a meta-analysis was conducted using the R statistical software v4.3, employing the metafor package. The objective was to quantitatively synthesize the association between *BAP1* mutations and staining and patient survival. The Hazard Ratios (HR) provided by individual studies were transformed into logarithms of Hazard Ratios (logHR) to stabilize the variance and facilitate more efficient statistical computation. Confidence intervals for the logHRs were calculated using the standard formula based on the delta method, which involves transforming the original study’s HR confidence intervals.

To assess heterogeneity among the studies, the I^2^ statistic was used, which measures the percentage of total variation across studies that is due to heterogeneity rather than chance. An I^2^ value over 50% is considered indicative of moderate to high heterogeneity. The DerSimonian–Laird method (DL method) was employed to estimate random effects, which was appropriate in the presence of heterogeneity among studies. Additionally, separate analyses were conducted for each group defined by the type of *BAP1* anomaly (mutation vs. stain). Each analysis was visualized using forest plots, which provide a graphical representation of each individual study and the aggregate estimate with their respective confidence intervals. This allowed for direct visual interpretation of the effect size and variability among studies.

## 3. Results

### 3.1. Literature Search and Study Characteristics

**Figure 1:**
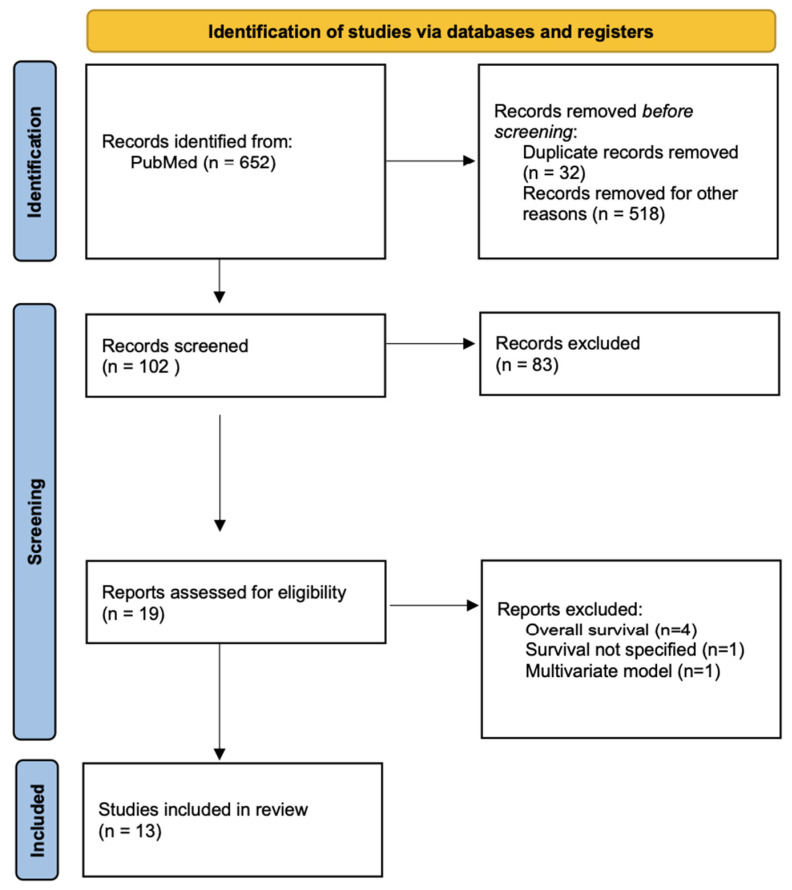
PRISMA flow diagram for study selection.

The initial database search conducted on 14 April 2024 retrieved a total of 823 titles (see Figure 1 for the PRISMA flow diagram). Thirty-two duplicates were detected and discarded. The remaining titles and abstracts were reviewed, and 689 records were excluded as they did not meet the eligibility criteria. The full-text versions of the remaining 102 articles were assessed, and 83 were excluded. Six studies were excluded after further consideration, as they included survival data as overall survival (n = 4), but they did not specify how survival was measured (n = 1) or included data from a multivariate model (n = 1). A total of 13 studies were included in the quantitative analysis.

A total of 13 retrospective cohort studies were included in the meta-analysis. Overall, 848 patients were included. The main characteristics of the studies reviewed are summarized in Table 1. We did not find any studies that met the inclusion criteria and provided information on *EIF1AX* mutations.

The assessment of bias risk, conducted with the Newcastle–Ottawa scale, is detailed in Table 2. All studies included in this meta-analysis were found to have a low risk of bias.

### 3.2. BAP1 Mutation and BAP1 Stain

**Figure 2:**
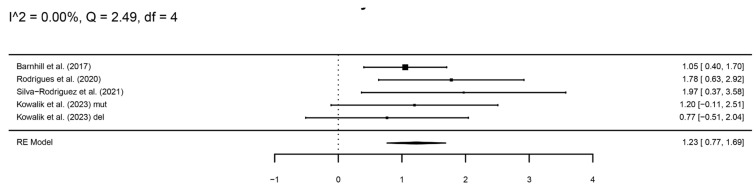
A forest plot showing the risk of metastasis in patients with a *BAP1* mutation [16,19,23,24].

A total of 76 patients with *BAP1* mutations and 82 controls were included. Kowalik et al. studied mutations (mut) and deletions (del) separately. The meta-analysis showed a significantly higher risk of metastasis in patients carrying a *BAP1* mutation compared to controls (log hazard ratio, logHR = 1.23, 95%CI 0.77–1.69) (Figure 2).

**Figure 3:**
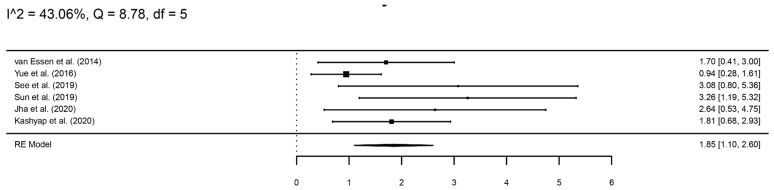
A forest plot showing the risk of metastasis in patients with negative BAP1 staining compared to those with a positive stain [14,15,17,18,20,21].

Nuclear BAP1 immunohistochemistry was negative in 200 patients and positive in 199 patients. The meta-analysis showed a significantly higher risk of metastasis in the group with negative BAP1 staining compared to positive staining (log hazard ratio, logHR = 1.85, 95%CI 1.10–2.60) (Figure 3).

**Figure 4:**
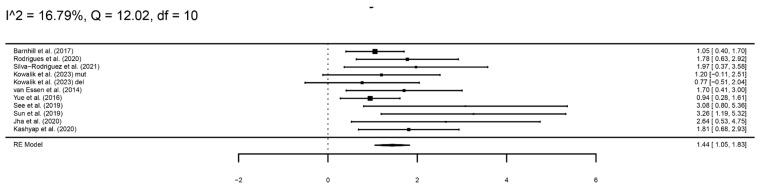
A forest plot showing a global analysis of *BAP1* mutation or negative nBAP1 staining compared to patients with negative mutation or positive staining [14,15,16,17,18,19,20,21,23,24].

A global analysis combining *BAP1* mutation and nBAP1 immunohistochemistry was performed (Figure 4), showing a significantly higher risk of metastasis in patients with either a *BAP1* mutation or negative nBAP1, compared to those without the mutation or positive immunohistochemistry (log hazard ratio, logHR = 1.44, 95%CI 1.05–1.83).

### 3.3. GNAQ Mutation

**Figure 5:**
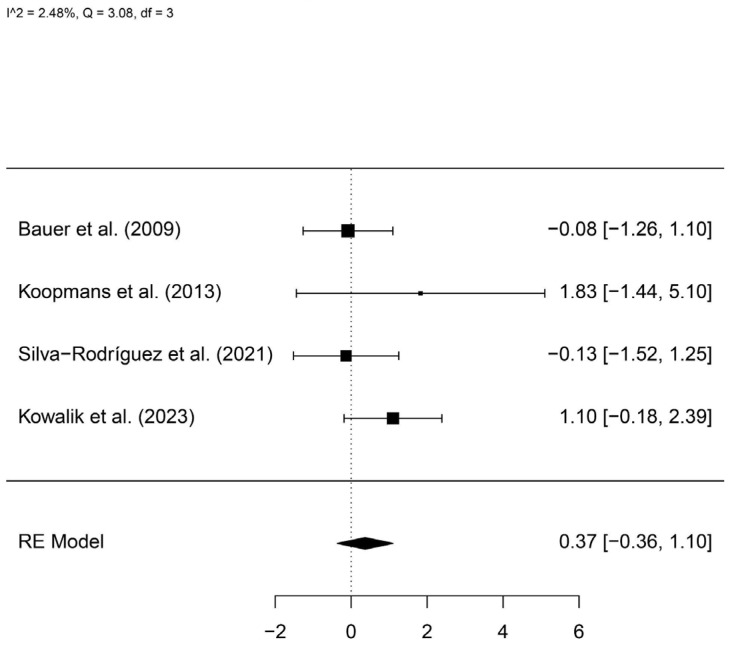
A forest plot comparing the risk of metastasis in patients with and without *GNAQ* mutations [12,13,23,24].

A total of 118 patients with *GNAQ* mutations and 75 controls were included. The meta-analysis showed no statistically significant difference between groups (log hazard ratio, logHR = 0.37, 95%IC −0.36–1.10) (Figure 5).

### 3.4. GNA11 Mutation

**Figure 6:**
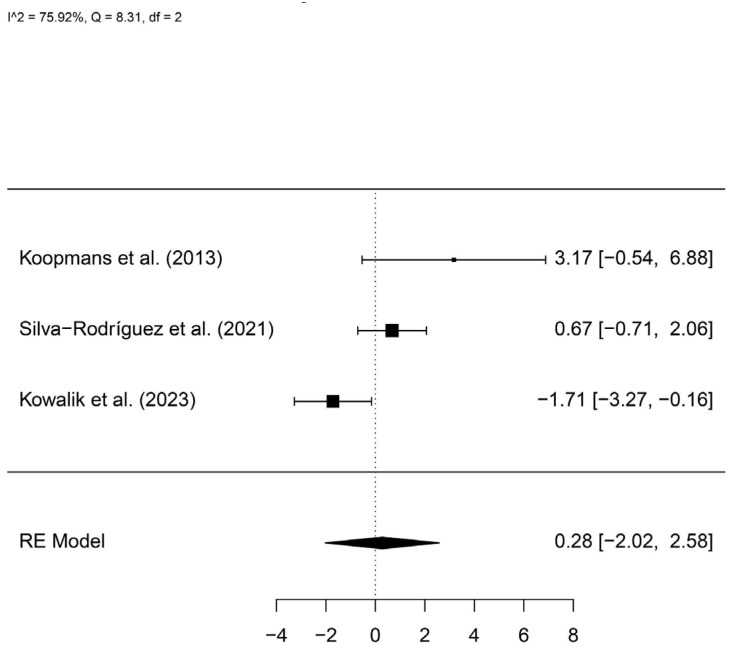
A forest plot comparing the risk of metastasis in patients with and without *GNA11* mutations [13,23,24].

A total of 66 patients with *GNA11* mutations and 46 controls were included. The meta-analysis showed no statistically significant difference between groups (log hazard ratio, logHR = 0.28, 95%IC −2.02–2.58) (Figure 6).

### 3.5. SF3B1 Mutation

**Figure 7:**
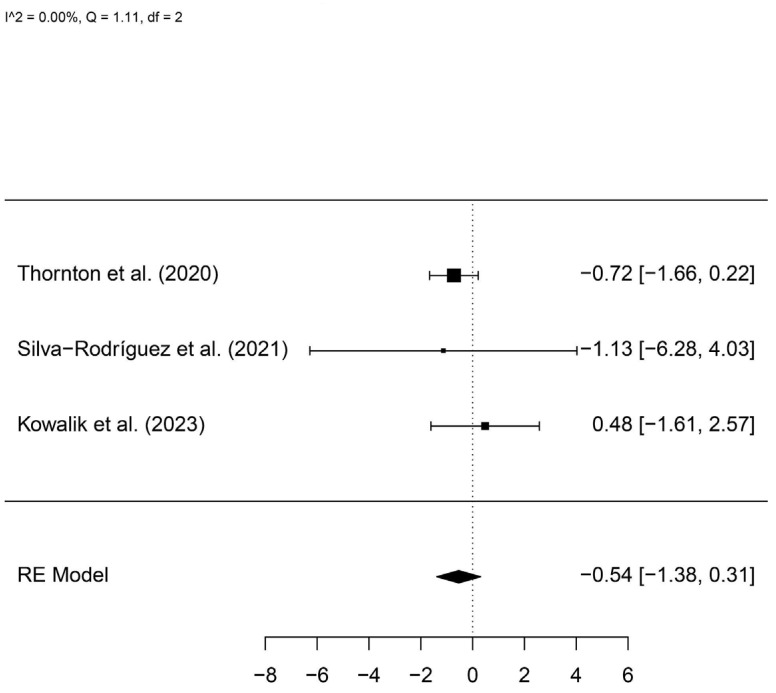
A forest plot comparing the risk of metastasis in patients with and without *SF3B1* mutations [22,23,24].

A total of 36 patients with *SF3B1* mutations and 174 controls were included. The meta-analysis showed no statistically significant difference between groups (log hazard ratio, logHR = −0.54, 95%IC −1.38–0.31) (Figure 7).

## 4. Discussion

The identification of driver mutations is fundamental in the field of cancer precision medicine, aiding in diagnosis, prognosis, and therapeutic choices for individual cancer patients. The literature reveals some variability regarding the prognostic outcomes of the main driver mutations in UM, which are found mostly in the *GNAQ*, *GNA11*, *EIF1AX*, *SF3B1*, and *BAP1* genes. Survival analysis varies across studies, with some concentrating on the risk of metastasis, others on disease-specific survival, and yet others on overall survival. Our meta-analysis found that the *BAP1* mutation predicts an increased risk of metastasis, whereas mutations in *GNAQ/GNA11* or *SF3B1* do not. To the best of our knowledge, this is the first meta-analysis assessing the prognostic value of driver mutations in UM. In our meta-analysis, we analyzed 13 retrospective cohort studies encompassing a total of 848 patients.

The BAP1 gene encodes the BRCA-1-associated protein 1 and is located on chromosome 3. Approximately 50% of UMs occur in patients with a biallelic inactivation of this gene, combining monosomy 3 (M3) and a deleterious somatic mutation in the second *BAP1* allele [25]. BAP1 is a deubiquitinating enzyme involved in several cellular processes, including transcriptional regulation, DNA repair, and metabolism, and it exhibits tumor suppressor activity in cancer cells [26]. *BAP1* loss may also help tumor cells evade immune detection, thereby promoting metastasis [27]. This meta-analysis found a similar risk of metastasis in patients with *BAP1* mutations (logHR = 1.23 [0.77–1.69]) and those with a negative BAP1 IHC (logHR = 1.85 [1.10–2.60]). This aligns with existing research, which shows that *BAP1* has been more extensively investigated than other driver mutations and is typically linked to a worse prognosis compared to other mutations. The concordance of BAP1 staining on immunohistochemistry (IHC) with *BAP1* mutation status in UM is well-documented, highlighting the utility of BAP1 IHC as a more accessible and economical diagnostic and prognostic marker. Studies show that negative nuclear BAP1 (nBAP1) staining typically corresponds with the presence of *BAP1* mutations [28,29]. Koopmans et al. report a sensitivity of 88% and specificity of 97% for IHC in detecting BAP1 expression [30]. However, in ambiguous cases, genetic analysis of the tissue for *BAP1* status is still recommended [31], as a non-functional BAP1 protein may create a false impression of normal protein levels based solely on IHC [18,32]. Next-generation sequencing (NGS) has become the standard for detecting BAP1 mutations and is the main method used in the studies represented in this meta-analysis. However, some alterations may be missed by some NGS algorithms, and assembly-based methods are required to identify them correctly [33]. Van de Nes et al. revealed that 14% of M3-UM have a hemizygous deletion of one or more *BAP1* exons rather than a sequence mutation. These alterations often go undetected by Sanger sequencing due to the presence of non-tumor DNA, necessitating gene dosage analysis for a complete mutation profile [29].

Mutations in G-protein subunits, specifically *GNAQ* and *GNA11*, are prevalent in uveal melanoma, occurring in approximately 85% of cases in a mutually exclusive pattern [23,34,35]. The *GNAQ* and *GNA11* genes produce proteins that belong to the Q class of G-protein alpha subunits, which play a role in transmitting signals from G-protein coupled receptors (GPCRs) to downstream effectors [36]. Mutations in *GNAQ* and *GNA11* genes cause continuous activation of GPCR signaling, implicated in promoting cell growth, tumor cell invasion, and drug resistance in UM. Such mutations occur early in the development of UM and are present even in the initial stages, including benign melanocytic lesions [34,37]. However, their association with survival is not clear. Our meta-analysis showed that mutations in *GNA11* or *GNAQ* did not correlate with a higher risk of metastasis. Of the four studies included in the meta-analysis, which provided information on *GNA11/GNAQ* status, only Kowalik et al. found better survival in the *GNA11* group and no difference in survival in *GNAQ* tumors [24]. The type of mutation might contribute to the differences among studies. Terai et al. suggested that distinct mutation patterns in *GNAQ* and *GNA11*, specifically Q209P vs. Q209L, could be more indicative of survival outcomes than the presence of mutations in *GNAQ* and *GNA11* themselves [38]. García-Mulero et al. found differences in immune system activation and infiltration between Q209P and Q209L tumors, although this did not translate to differences in survival [39]. Similar results were described by Schneider et al. Piaggio et al. found no difference in disease-specific survival among patients with *GNA11* vs. *GNAQ* tumors in an overall analysis, although *GNA11* appeared to have a worse prognosis compared to *GNAQ* in M3 and *BAP1* mutated patients [40]. These studies could not be included in the meta-analysis as they provided hazard ratios, which included one type of mutation as the comparator instead of the absence of mutation. Further research with larger and well-defined groups is necessary to establish the prognostic significance of G-protein mutations.

The *SF3B1* gene plays a pivotal role in ensuring the correct processing of RNA transcripts into mature mRNA, as it encodes a component of the spliceosome, the crucial cellular machinery responsible for RNA splicing [41]. Mutations in *SF3B1* have been correlated with an intermediate potential to develop metastatic disease, occurring later than *BAP1* mutations, usually around 7 years after primary treatment [42,43]. Other studies have shown better survival rates in *SF3B1* mutated patients compared to wild-type patients [44]. This may be explained by certain prognostic features found in patients with *SF3B1* mutations, such as lower age at diagnosis and tumors exhibiting disomy 3, contrasting with *BAP1*-mutated tumors [43], and because these mutations are usually mutually exclusive [23,25]. Our meta-analysis showed there was no increase in metastatic risk in patients carrying *SF3B1* mutations. This result should be confirmed by including studies with longer follow-up times.

This meta-analysis has several limitations. It was carried out on published data instead of individual data, using a univariate hazard ratio for estimating the risk of metastasis, which ignored other variables and their influence on survival. Some data were not available in some patients, mainly due to the retrospective design of the studies included in this analysis. Moderate heterogeneity among studies with BAP1 stain may be due to different cut-off values. Other driver mutations, including *EIF1AX*, could not be analyzed in this study as hazard ratios for MFS were only provided by one author and used a multivariate model. The combination of chromosomal alterations (such as monosomy 3 or 8q gain) with mutations in driver genes could not be analyzed as most authors do not report segregated data. Most studies analyzed samples obtained from enucleated eyes, and thereby, their results may not reflect the features of smaller UMs, which are more often treated by globe-sparing techniques. The heterogeneity in reporting survival among studies also limited the number of studies that could be included in this meta-analysis. Larger cohorts should be included in future studies, enhancing the robustness and wider applicability of the results.

## 5. Conclusions

In summary, this is the first meta-analysis of driver mutations in uveal melanoma, showing that *BAP1* mutations are linked to an increased risk of metastatic disease in uveal melanoma, while no such association was observed with *GNAQ/GNA11* and *SF3B1* mutations. Our results confirm the comparability of BAP1 immunohistochemistry as a prognostic marker, as it offered a similar prognostic value as genetic analysis.

## Figures and Tables

**Table 1:** Summary of studies included in the meta-analysis. Age is expressed as mean and standard deviation or median and range in years. Follow-up in months (median and range). Molecular test: PCR: polymerase chain reaction, GEP: genome-wide gene expression profiling, IHC: immunohistochemistry, NGS: next-generation sequencing. HR: hazard ratio, CI: 95% confidence interval.

Author (Year)	Country	N	Age (Years)	Follow-Up (Months)	Treatment	Mutation/Stain	Molecular Test	N Mutation	N Controls	HR	Low CI	High CI
Bauer et al. (2009) [12]	Holland	75	62 (21–86)	56.1 (6.4–136.4)	Enucleation	*GNAQ*	PCR	40	35	0.92	0.24	2.55
Koopmans et al. (2013) [13]	Holland	92	62 (21–86)	74.9 (5.2–200.5)	Enucleation	*GNAQ*	PCR	46	6	6.22	0.07	48.52
						*GNA11*	PCR	40	6	23.86	0.08	133.45
van Essen et al. (2014) [14]	Holland	30	61.7 (28–84)	77.5 (14–155)	Enucleation	BAP1 -	IHC	14	14	5.5	1.5	20.1
Yue et al. (2016) [15]	China	171	48.6 (14–83)	63.4 (6.4–140.1)	Enucleation	BAP1 -	IHC	53	103	2.57	1.32	5.01
Barnhill et al. (2017) [16]	France	89	63 (25–92)	53 (2.4–132)	Enucleation	*BAP1*mut	GEP	39	36	2.864	1.494	5.49
See et al. (2019) [17]	USA	30	62 ± 16	30.3 ± 23	Enucleation	BAP1 -	IHC	19	11	21.7	2.2	210.1
Sun et al. (2019) [18]	Sweden and USA	47	63 ± 14	89 ± 98	Enucleation	BAP1 -	IHC	22	25	26	3.3	205.9
Rodrigues et al. (2020) [19]	France	43	58 (12–79)	66 (1.2–126.2)	Enucleation (42%), ProtonBeam, I-125 brachytherapy	*BAP1*del	GEP	19	24	5.91	1.89	18.54
Kashyap et al. (2020) [20]	India	69	40	Range 10–65	Enucleation	BAP1 -	IHC	37	13	6.1	1.98	18.77
Jha et al. (2020) [21]	India	69	Range 17–92	Range 10–65	Enucleation	*BAP1*del	IHC	46	23	13.97	1.37	93.46
Thornton et al. (2020) [22]	UK	117	64 (16–87)	65 (0–132)	Enucleation (66%), local resection 10%), endoresection (1%), Ruthenium Brachytherapy (14%); ProtonBeam (9%)	*SF3B1*	NGS	25	119	0.486	0.19	1.241
						*BAP1*mut	NGS	50	63	6.536	3.095	13.804
Silva-Rodríguez et al. (2021) [23]	Spain	46	68 (40–91)	31.2 (6.1–51.9)	Brachytherapy + endoresection or enucleation	*BAP1*mut	NGS	8	29	7.17	1.44	35.69
						*GNAQ*	NGS	24	22	0.876	0.219	3.506
						*GNA11*	NGS	16	30	1.964	0.491	7.859
						*SF3B1*	NGS	10	36	0.324	0	29.989
Kowalik et al. (2023) [24]	Poland	20	62.0 (53.8–68.2)	NA	Enucleation	*BAP1*mut	NGS	9	11	3.31	0.89	12.23
						*BAP1*del	NGS	9	11	2.15	0.6	7.73
						*GNAQ*	NGS	8	12	3.01	0.83	10.88
						*GNA11*	NGS	10	10	0.18	0.038	0.86
						*SF3B1*	NGS	1	19	1.62	0.2	13.11

**Table 2:** Newcastle–Ottawa scale for risk of bias assessment.

	Study Design	Selection	Comparability	Outcomes	Total Score
Bauer et al. (2009) [12]	cohort	3	1	2	6
Koopmans et al. (2013) [13]	cohort	2	1	3	6
van Essen et al. (2014) [14]	cohort	3	1	3	7
Yue et al. (2016) [15]	cohort	2	2	3	7
Barnhill et al. (2017) [16]	cohort	2	1	3	6
See et al. (2019) [17]	cohort	3	1	3	7
Sun et al. (2019) [18]	cohort	3	1	3	7
Jha et al. (2020) [21]	cohort	3	2	3	8
Kashyap et al. (2020) [20]	cohort	3	1	2	6
Rodrigues et al. (2020) [19]	cohort	3	2	3	8
Thornton et al. (2020) [22]	cohort	3	1	3	7
Silva-Rodríguez et al. (2021) [23]	cohort	3	1	3	7
Kowalik et al. (2023) [24]	cohort	3	1	2	6

## Data Availability

Data are available upon request.

## Supplementary Materials

The following supporting information can be downloaded at: https://www.mdpi.com/article/10.3390/cancers16142510/s1, Figures S1 and S2: Funnel and Galbraith plots for *BAP1*; Figures S3 and S4: *GNA11*; Figures S5 and S6: *GNAQ*; Figures S7 and S8: *SF3B1* genes.
