# Supplementary figures and images for: Impact of Driver Mutations on Metastasis-Free Survival in Uveal Melanoma: A Meta-Analysis
Source: Cancers (Basel). 2024 Jul 10;16(14):2510. doi: 10.3390/cancers16142510 (PMC11274588; doi:10.3390/cancers16142510)

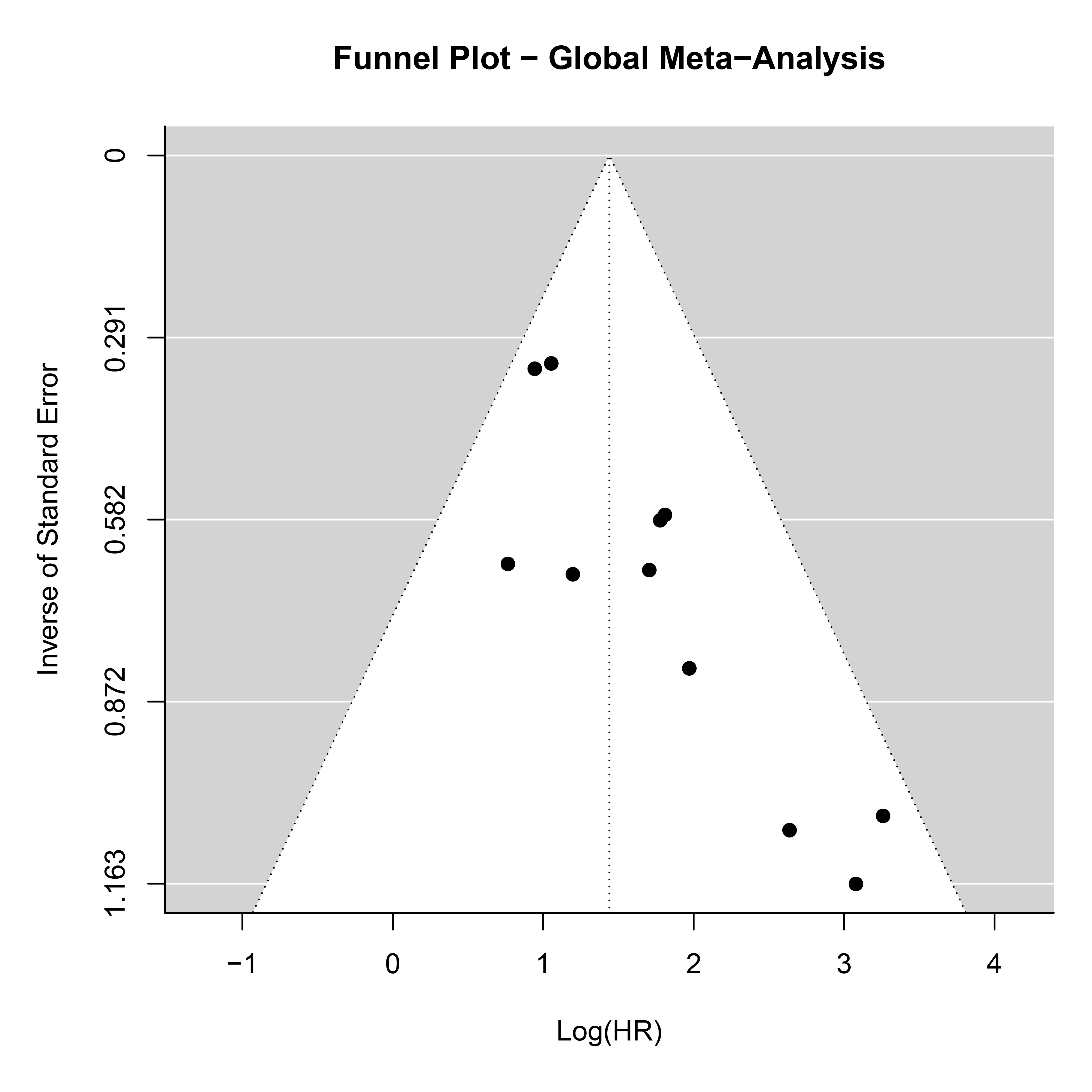

Supplement: Supplementary file 1 [file cancers-16-02510-s001.zip › s1-Funnel and Galbraith plots for BAP1.jpg]

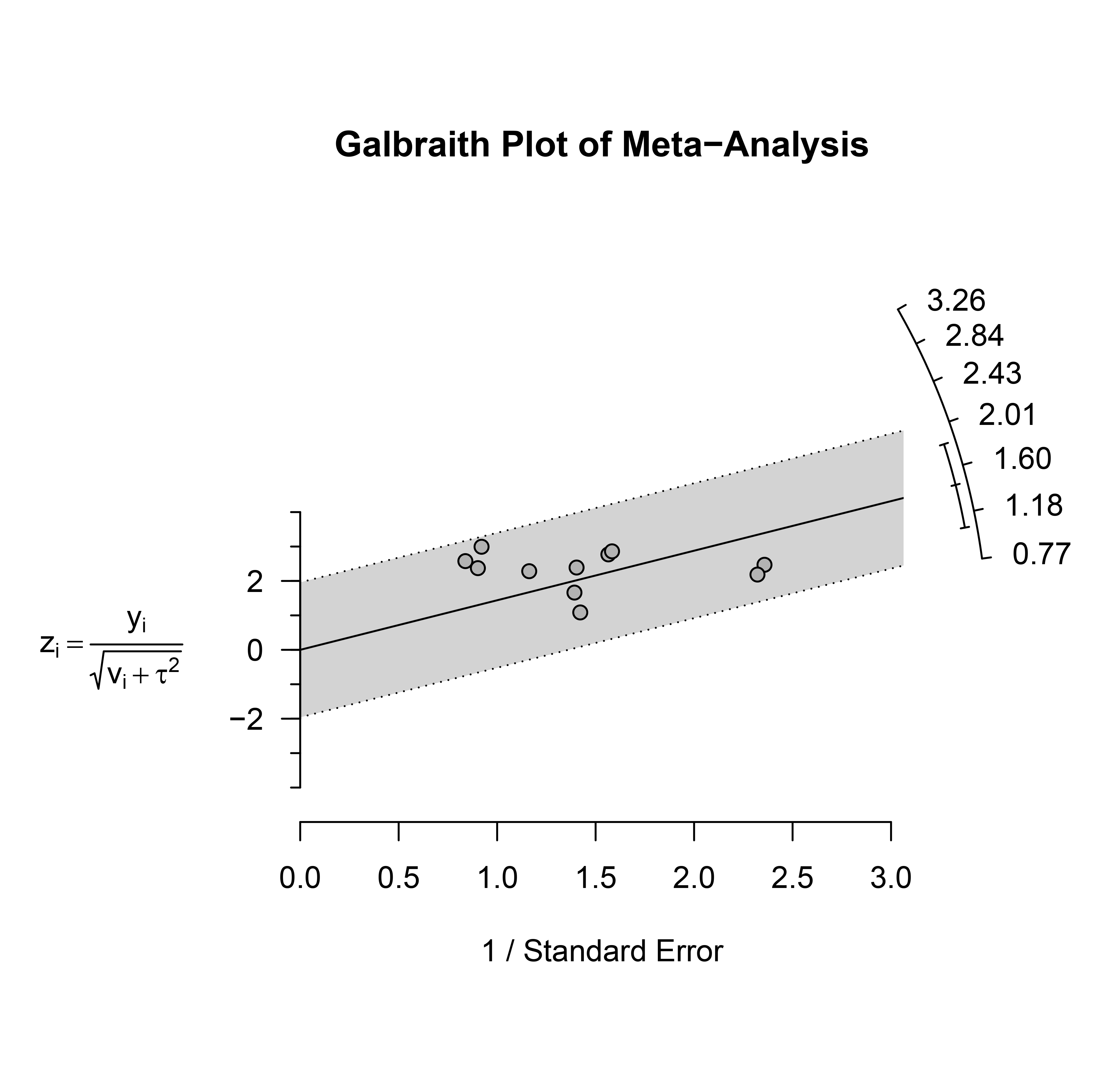

Supplement: Supplementary file 1 [file cancers-16-02510-s001.zip › s2-Funnel and Galbraith plots for BAP1.jpg]

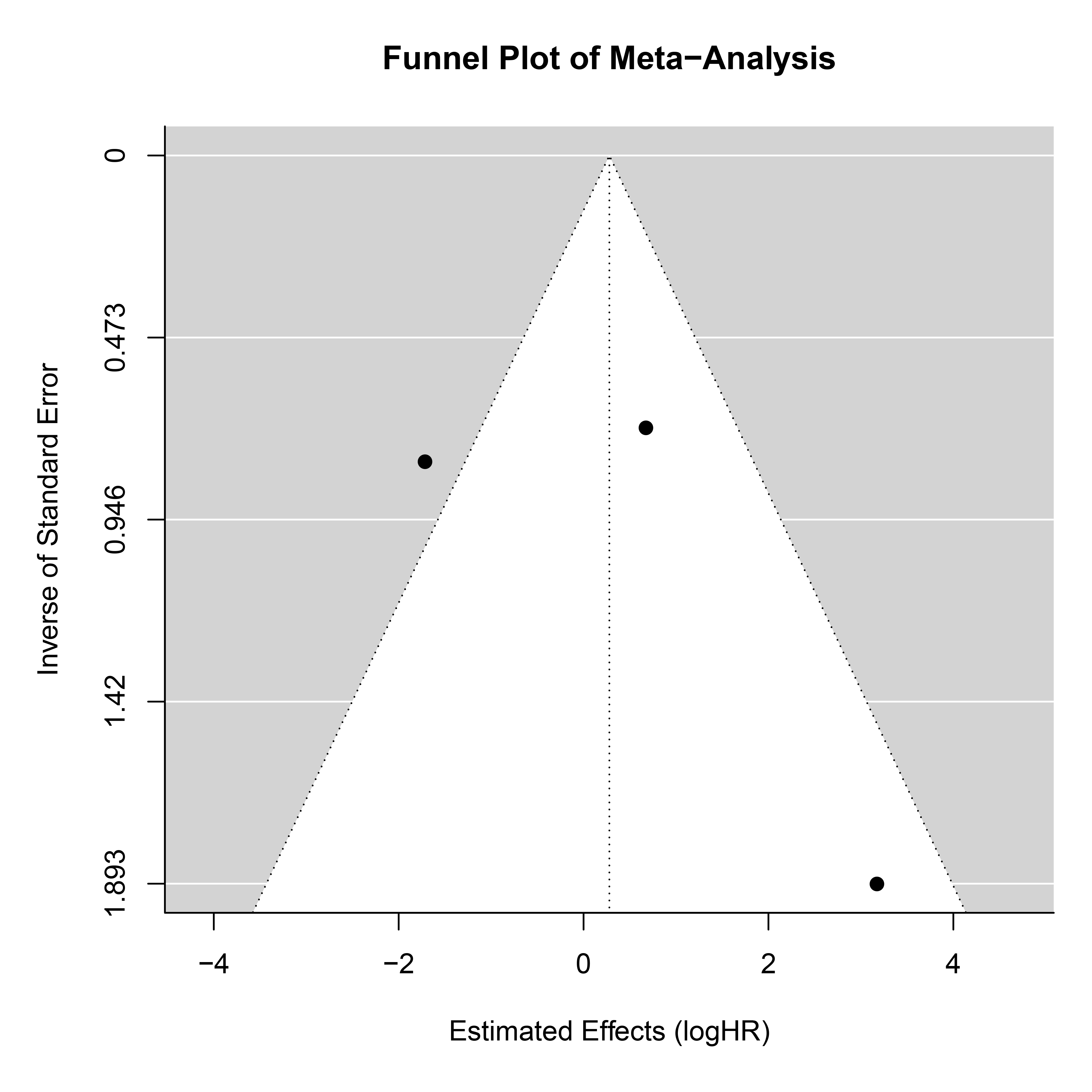

Supplement: Supplementary file 1 [file cancers-16-02510-s001.zip › s3-GNA11.jpg]

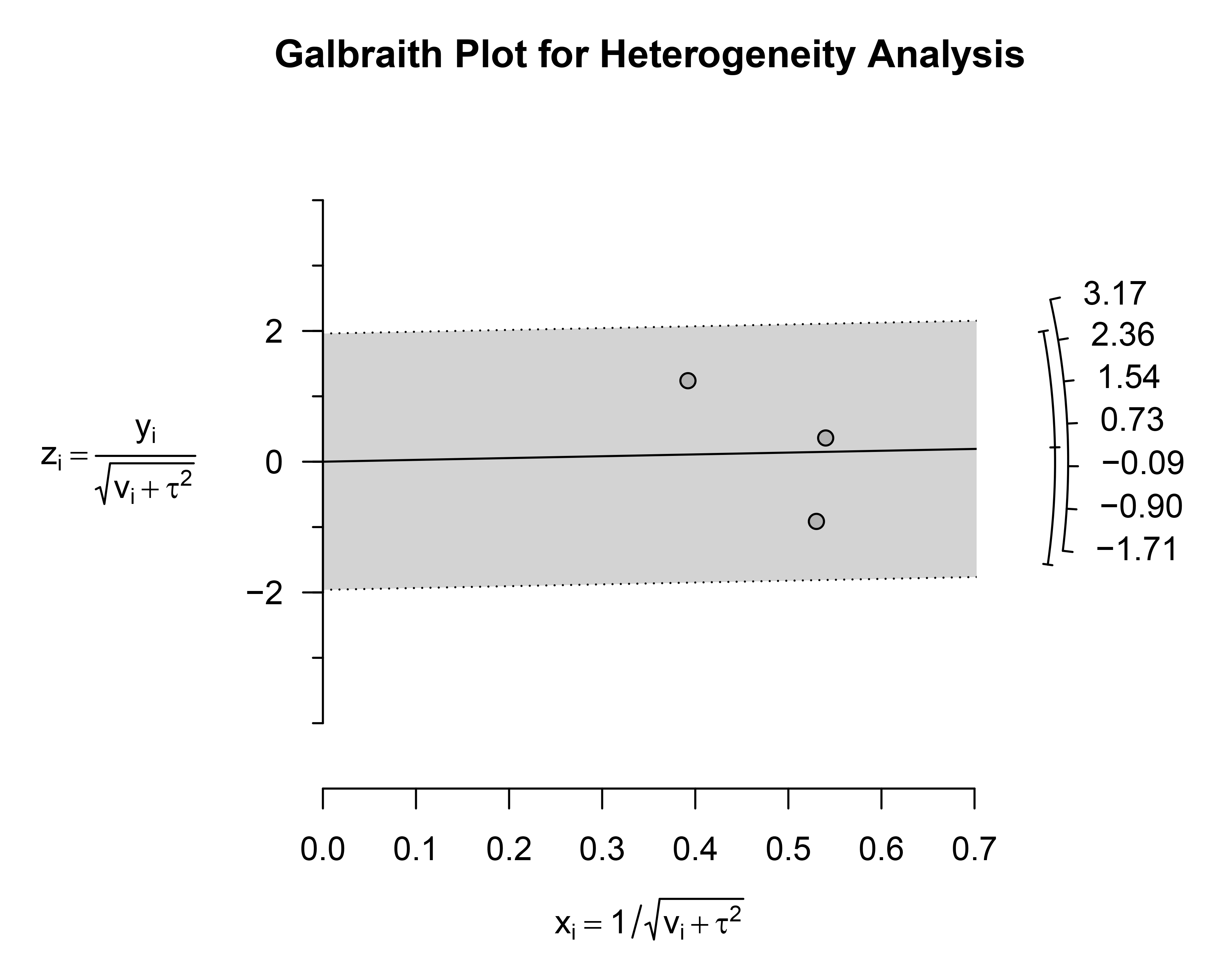

Supplement: Supplementary file 1 [file cancers-16-02510-s001.zip › s4-GNA11.jpg]

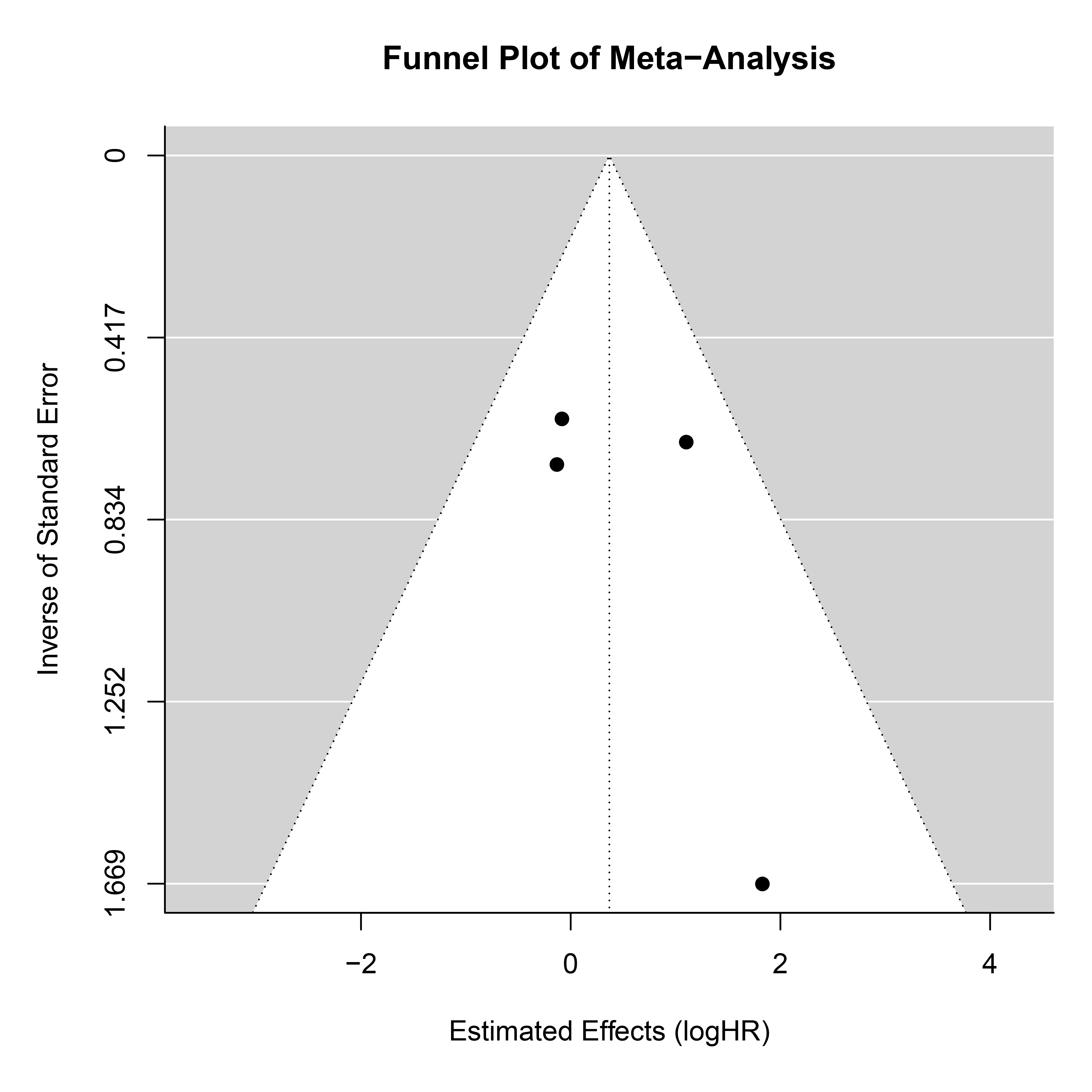

Supplement: Supplementary file 1 [file cancers-16-02510-s001.zip › s5-GNAQ.jpg]

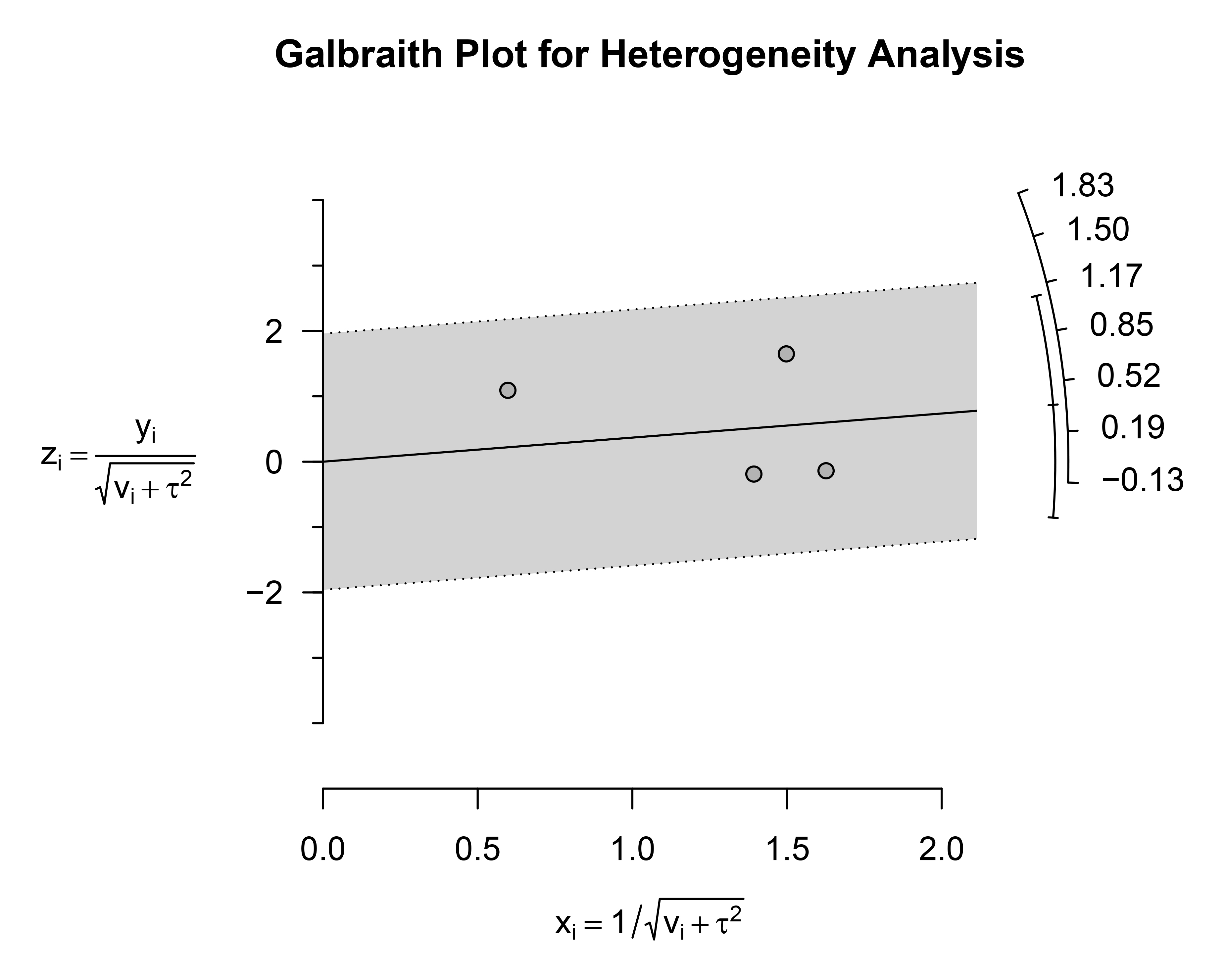

Supplement: Supplementary file 1 [file cancers-16-02510-s001.zip › s6-GNAQ.jpg]

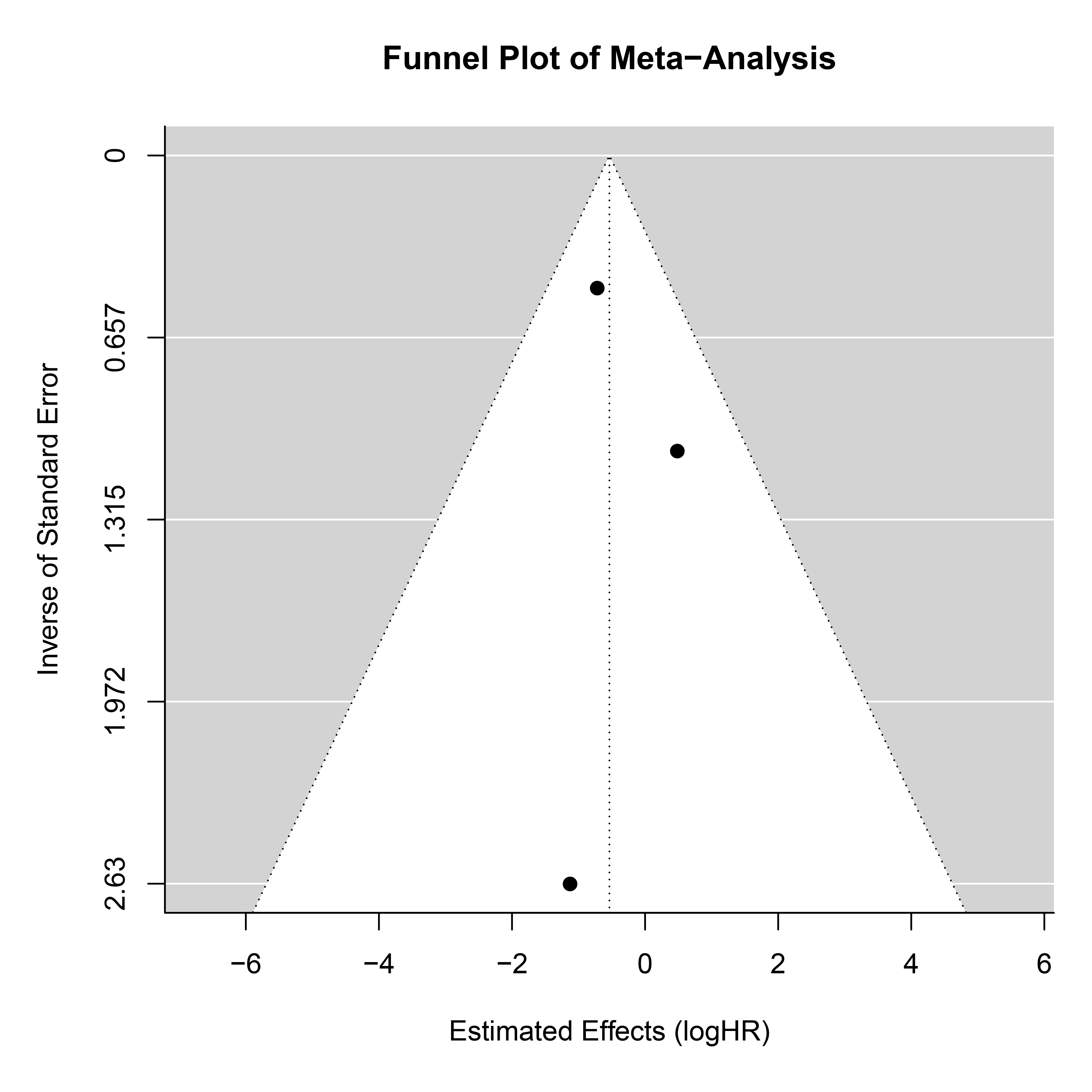

Supplement: Supplementary file 1 [file cancers-16-02510-s001.zip › s7-SF3B1 genes.jpg]

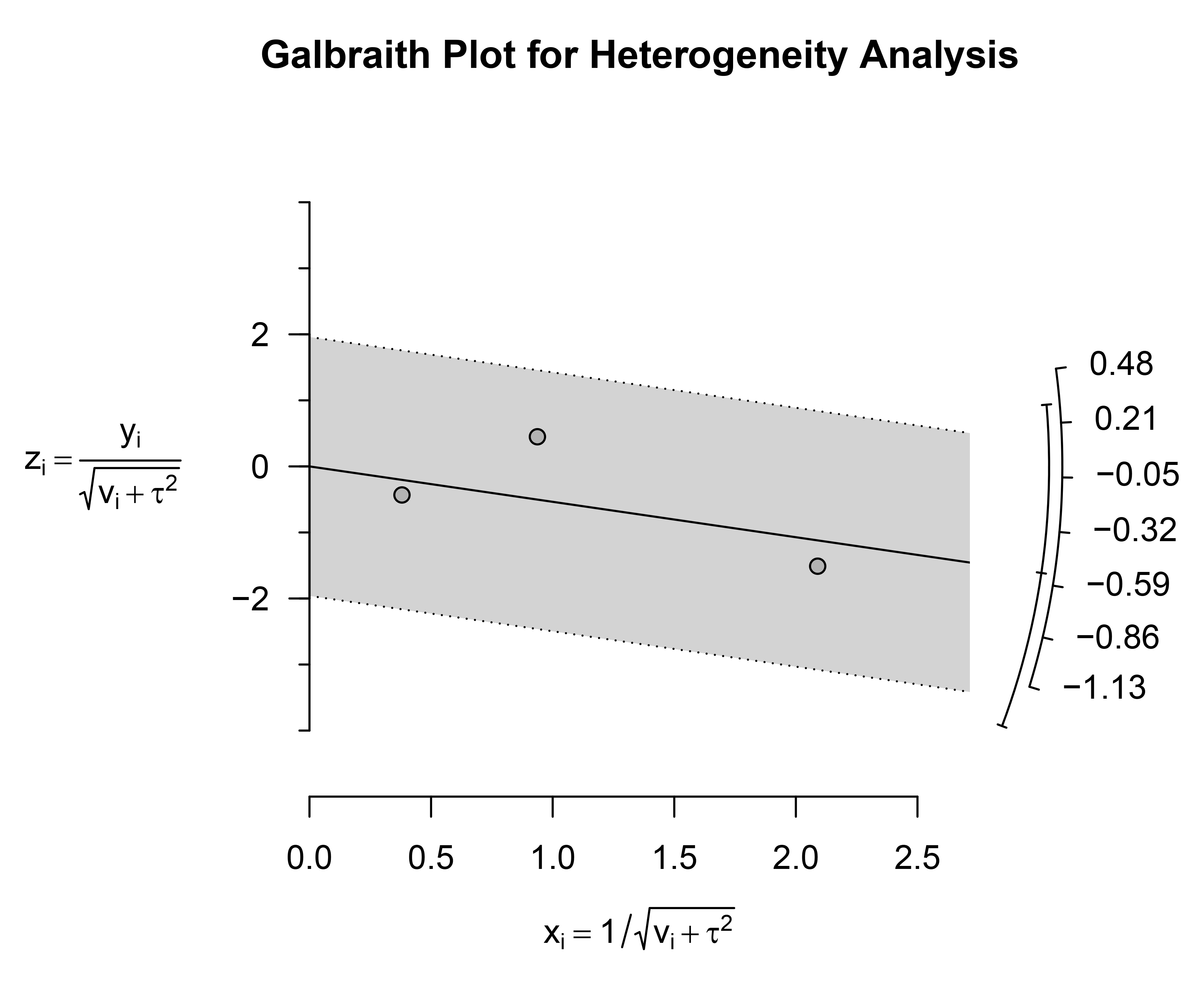

Supplement: Supplementary file 1 [file cancers-16-02510-s001.zip › s8-SF3B1 genes.jpg]
